# Editorial: Down syndrome: Genetic and epigenetic influences on this multi-faceted condition

**DOI:** 10.3389/fgene.2023.1163133

**Published:** 2023-03-24

**Authors:** Sujay Ghosh, Colleen Jackson-Cook, Nishant Singhal, Subhra Prakash Hui

**Affiliations:** ^1^ Department of Zoology, University of Calcutta, Kolkata, India; ^2^ Department of Pathology, Virginia Commonwealth University, Richmond, VA, United States; ^3^ National Centre for Cell Science, Pune, Maharashtra, India

**Keywords:** Down syndrome, trisomy 21, mouse model, pluripotent stem cells, hearing disorders, maternal age, neurogenesis

Down syndrome is caused due to a trisomic imbalance of human chromosome 21 and has been associated with more than 80 clinical symptoms. The clinical symptoms include short stature, muscle hypotonia, atlantoaxial instability, reduced neuronal density, cerebellar hypoplasia, intellectual disability, and congenital heart defects [Epstein et al. 1991; Am J Hum Genet: 49 (1):207-35]. In addition to traits that are typically present in infancy/early childhood, individuals with DS are also more likely to develop certain health conditions, like hypothyroidism, autoimmune diseases, obstructive sleep apnoea, epilepsy, hearing and vision problems, haematological disorders (including leukaemia), recurrent infections, anxiety disorders, and early-onset Alzheimer’s disease. Given the phenotypic variation associated with Down syndrome, studies on this condition could provide information to better understand the causes of many congenital phenotypes and their clinical management. Research advances have provided insights about the complexity of the genetic/epigenetic alterations underlying the clinical outcomes associated with Down syndrome. These changes reflect direct effects arising from triplosensitivity of genes on chromosome 21, as well as “cross talking” with disomic genes on other chromosomes, including those involved in epigenetic regulation. Allelic polymorphisms of genes contribute to further variability in the manifestation of phenotypic traits associated with Down syndrome. In addition to research on clinical subjects, animal models, as well as induced pluripotent stem cells (iPSCs) and organoids, have offered opportunities to unravel the causes and consequences of a trisomy 21 gene dosage imbalance and provided clues for designing future therapeutic strategies to alleviate symptoms of health conditions acquired by people with Down syndrome. The models of mice, *Drosophila*, and *Caenorhabditis elegans* have offered opportunities to gain insights into genotype–phenotype correlations for Down syndrome. Recently developed iPSC lines for trisomic cells have helped unravel the transcriptomic and metabolomic states of single trisomic cells, using multi-omics approaches. The creation of new model systems and new isogenic trisomic/disomic iPSC lines hold further promise to open new avenues for designing therapeutic strategies. This Research Topic of Frontiers in Genetics highlights contemporary developments and challenges in the field of trisomy 21 research. In their original research article entitled “Hearing impairment in murine model of Down syndrome,” Chen et al. described their experiments on a Down syndrome mouse model, Dp(16)1Yey, to characterise the structural and functional alterations in the auditory system, leading to hearing impairment. They reported reduced outer hair cell function, smaller tympanic diameter and oval window, shorter middle ear space, shorter cochlear basal membrane, and altered responses from the cochlear auditory nerve among the Down syndrome-model mice compared to the wild-type controls. The investigators also suggested that future research projects will be completed to better define factors leading to conductive hearing impairment in some people with Down syndrome.

In the original research article entitled “Biphasic cell cycle defect causes impaired neurogenesis in Down syndrome,” Sharma et al. studied the causes of impaired neurogenesis using iPSCs that were derived from human trisomic cells, as well as iPSCs derived from the TS65Dn mouse model of Down syndrome. The authors characterised reduced proliferation of Down syndrome model cells at the early phase, followed by increased proliferation at the late phase of the neurogenic stage, when compared to control cell lines. Interestingly, both events may account for reduced neurogenesis; for example, while reduced proliferation of the trisomic model cells results in generation of less neural progenitor cells, an elevated rate of division at a later stage(s) causes delayed post-mitotic generation of neurons in model systems/people with Down syndrome. Furthermore, RNAseq analysis of late-phase Down syndrome model progenitor cells revealed upregulation of S-phase-promoting regulator genes and downregulation of the genes of the BAF chromatin remodelling complex. The ChIPseq analysis of late-phase neural progenitors revealed aberrant PAX6 binding to the downstream genes. Thus, these findings indicated, for the first time, that impaired neurogenesis in DS is due to biphasic proliferative defects, as opposed to either reduced proliferation or increased proliferation, as proposed previously. Additionally, the authors suggested that aberrant regulation of genes and altered PAX6 transcription factor binding could be a cause of defective neurogenesis associated with Down syndrome.

In their original research article entitled, “Incidence of Down Syndrome by maternal age in Chinese population,” Song et al. completed a retrospective study to estimate the incidence of trisomy 21/partial trisomy 21 in second-trimester pregnancies of Chinese women, based on data collected from maternal serum screening. The incidence was calculated for each of three maternal age groups: (1) 26 years of age or younger; (2) 27–33 years of age; or (3) 34 years of age or older. As expected, the rate of trisomic pregnancies was significantly higher among the 34 years of age or older group of women (incidence of 2.07 per 1,000 pregnancies). The younger women (26 years of age or younger) had a significantly higher risk for a trisomy 21 pregnancy (incidence of 0.67 per 1,000 pregnancies), compared to women aged 27–33 years (incidence of 0.29 per 1,000 pregnancies). This report provides a more recent assessment of pregnancy rates for trisomy 21/partial trisomy 21 among Chinese women.

In a comprehensive review entitled, “Enhanced GIRK2 channel signaling in Down syndrome: A feasible role in the development of abnormal nascent neural circuits,” Kleschevnikov described how a trisomic imbalance for the *KCNJ6* gene (localised to chromosome 21 in humans) leads to overexpression of a potassium channel subunit (GIRK2). The author further suggested that this gene pathway could contribute to cognitive decline and altered synaptic plasticity in people with Down syndrome. This review focused on different studies using Ts65Dn mice that exhibited overexpression of Girk2 and possible therapeutic strategies to restore the wild-type expression pattern. The author concluded that correction of gene dosage in the neonatal brain, by time limited pharmacological or genetic interventions, may improve cognitive function in people with Down syndrome.

We hope this special issue will draw the attention of the Down syndrome research with community, including investigators who are engaged in both clinical and pre-clinical models. The readers will learn about various research initiatives that are being taken, across the globe, to improve the quality of life for individuals with Down syndrome.

